# 16 Years (2006–2021) of Surface Ozone Measurements in Córdoba (Southern Spain): Trends and the Impact of the COVID-19 Lockdown

**DOI:** 10.3390/ijerph192316210

**Published:** 2022-12-03

**Authors:** Miguel A. Hernández-Ceballos, Alberto Jiménez-Solano, Julio Torres-Fernández

**Affiliations:** Department of Physics, University of Cordoba, 14071 Cordoba, Spain

**Keywords:** surface ozone, ozone trends, COVID-19 lockdown, southern Iberian Peninsula, Guadalquivir valley

## Abstract

Surface ozone concentrations (O_3_) during the period 2006–2021 are analysed at Córdoba city (southern Iberian Peninsula) in suburban and urban sampling sites. The aims are to present the levels and temporal variations, to explore trends and to quantity the variation in O_3_ concentrations in the context of the COVID-19 lockdown. The O_3_ means are higher in the suburban station (62 µg m^−3^ and 51.3 µg m^−3^), being the information level threshold only exceeded twice during this period. The daily evolution shows a maximum at about 17:00 UTC, whereas the minimum is reached at about 9:00 UTC, with higher levels in the suburban station. The seasonal evolution of this daily cycle also presents monthly differences in shape and intensity between stations. The trends are analysed by means of daily averages and daily 5th and 95th percentiles, and they show a similar increase in all of these parameters, with special emphasis on the daily P95 concentrations, with 0.27 µg m^−3^ year^−1^ and 0.24 µg m^−3^ year^−1^. Finally, the impact of the COVID-19 lockdown shows a decline in O_3_ concentrations over 10%.

## 1. Introduction

Ozone is a short-lived trace gas that, at the surface, is a pollutant, and hence, at high levels, it is harmful to both human and plant health [1]. Ozone can have a tropospheric and stratospheric origin. While it is formed naturally in the stratosphere and then transported to surface layers, in the troposphere, it is formed by the interaction of ultraviolet light with chemicals (nitrogen oxides, nonmethane volatile organic compounds, methane or carbon monoxide) emitted by natural and anthropogenic sources [2].

Globally, the spatial and temporal distribution of tropospheric ozone, also known as surface-level ozone (O_3_), is largely analysed, either by observations or numerical simulations [3,4,5]. In this sense, O_3_ concentrations and trends widely depend on the location of the sampling site. For instance, O_3_ has decreased since 2000 at coastal stations and at mountain sites [6,7], while at non-urban stations, it presents increasing and decreasing trends depending on the season [8]. Considering this spatial and temporal variability, the greater number of sampling sites at specific locations evaluated, the better the picture of the O_3_ trends we have. 

**Figure 1:**
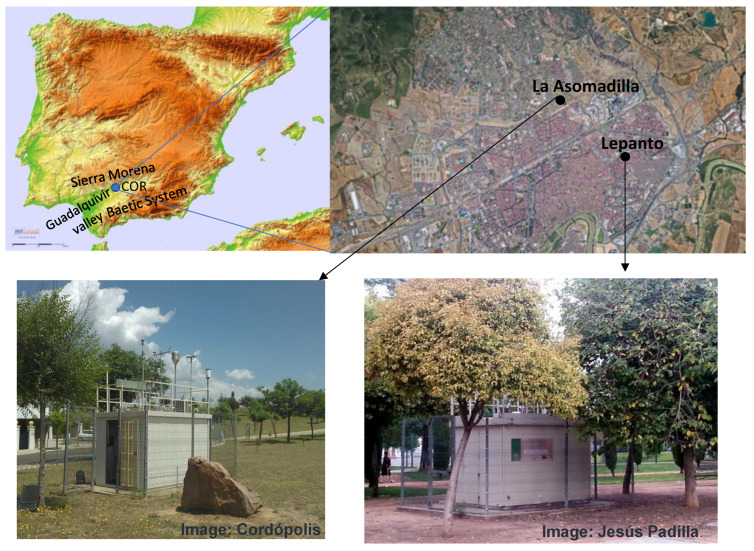
Study area and the location of both air quality stations in Córdoba city.

In this context, many studies have pointed out that the western Mediterranean, due to its morphological characteristics and the development of mesoscale circulations [9], is a region highly affected by O_3_ pollution, with accumulated concentrations exceeding the threshold levels indicated by the World Health Organization “https://www.who.int/news-room/fact-sheets/detail/ambient-(outdoor)-air-quality-and-health (accessed on 5 September 2022)”. Within this region, the Guadalquivir Valley is one of three major O_3_ hotspots in Spain [10]. This valley (650 km and 57,000 km^2^) crosses the Andalusia region (southern Iberian Peninsula) along the southwest–northeast axis (Figure 1). The location within the valley of significant emission sources of both biogenic (Doñana National Park) and anthropogenic (e.g., Seville metropolitan area, Huelva, Cádiz, Jaén and Córdoba cities and the industrial area of Huelva) together with high levels of temperature and solar radiation play a key role in driving the occurrence of O_3_ events. Many authors have investigated the temporal and spatial variability of O_3_ in this specific region and the relationship with synoptic weather, and local and regional circulation patterns [11,12].

Previous studies [13,14] found persistent and high O_3_ concentrations in monitoring stations located in Córdoba city, which is a medium-sized city (approximately 340,000 inhabitants) placed in the middle of the valley and close to the Sierra Morena Mountain chain to the north. O_3_ concentrations in Córdoba were recently used to understand the local and remote contributions to O_3_ in a pollution event in the Guadalquivir valley [10], and O_3_ peak concentrations in Córdoba were included in [15] to understand the phenomenology of high O_3_ pollution in the Guadalquivir valley. However, to the authors’ knowledge, this is the first time that O_3_ concentrations in Córdoba have been solely studied. Doing this, the present analysis and results complement the previous analysis of O_3_ performed in this region, and at the same time, will allow the investigation of the usefulness of control strategies implemented in cities to improve air quality. 

The present paper, hence, characterises and analyses changes of near-surface O_3_ concentrations in Córdoba city. To investigate the spatial and temporal variability at different locations within the city, we used 16 years (2006–2021) of continuous hourly O_3_ measurements at two surface monitoring stations. The possibility to work with this long time series ensured a large and statistically representative sample, hence allowing the possibility to determine long-term changes in the O_3_ concentrations at this specific site. In this sense, O_3_ trends during this period are studied based on daily averages and daily 95th and 5th percentiles [16].

This O_3_ time series covers the COVID-19 health crisis, which has been extensively analysed in terms of air quality all around the world [17,18]. In Spain [19], an analysis was performed on how O_3_ concentrations changed during this period at different monitoring stations, although Córdoba city was not considered in the study. Therefore, the present study is also the first analysis describing the impact of this special period on the O_3_ concentrations in this city. 

In general, the following research issues are addressed:

− To compare between O_3_ concentrations at two monitoring stations placed in different urban environments;
− To investigate the yearly, seasonal and daily variability of O_3_ concentrations in Córdoba during the period 2006–2021;
− To quantify the trends for the O_3_ levels;
− To quantify changes in the O_3_ concentrations associated with the COVID-19 period.

## 2. Description of Data

The analysed data consist of 16 years of continuous measurements with one hour resolution of O_3_ concentrations. These O_3_ data were collected at two official monitoring stations of the Andalucía regional government locates in Córdoba city. These two stations are Asomadilla (37.90 N, −4.77 W, 152 m above sea level) and Lepanto (37.89 N, −4.76 W; 119 m above sea level) (hereafter, ASO and LEP). While ASO is classified as a suburban background, LEP is an urban background. ASO is in the fairly open Asomadilla Park, which is the largest park in Córdoba and is located 2 km north of the centre the city, while LEP is located in a small city park surrounded by a built-up urban area (residential and commercial area) (Figure 1).

**Figure 2:**
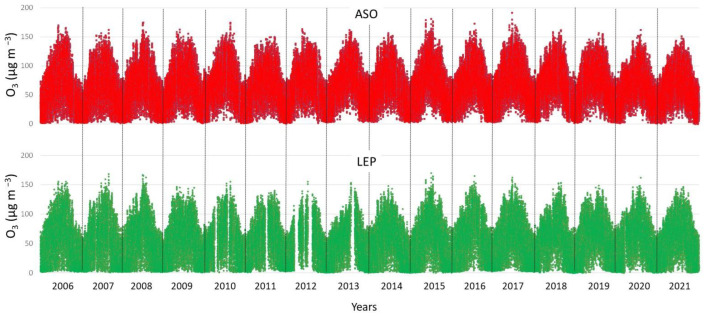
Hourly evolution of O_3_ concentrations in Asomadilla and Lepanto monitoring stations during the period 2006–2021.

Concentrations were recorded at the air quality stations every hour during the period 2006–2021. Each value represents the concentration obtained with the start time of sampling. Hence, the 24-hourly concentrations are from 0:00 to 23:00 h, in Coordinated Universal Time (UTC). Working with data, it is ideal to acquire them continuously without gaps; however, this is almost impossible due to power failure or other technical problems. These gaps in the time series can be seen in Figure 2. The O_3_ hourly data covers 96% and 92% of the total O_3_ values during the sampling period (2006–2021). From this set of hourly measures, the quality criterion of 75% was applied to calculate daily values (means, 95th and 5th percentiles), and, then monthly and yearly values, i.e., at least 75% of the hourly and daily records.

## 3. Results

### 3.1. O_3_ Characterisation in Córdoba City 

In this section, we illustrate the O_3_ concentrations measured in Córdoba and present similarities and differences within the city. Figure 2 displays the hourly evolution of O_3_ surface concentrations at ASO and LEP during the study period 2006–2021. There was a high and positive correlation between both (0.94 at the 0.05 significance level), as was expected between two stations in the same city. The means for the whole period were 62.0 µg m^−3^ in ASO and 51.3 µg m^−3^ in LEP. The highest hourly concentration was recorded in ASO, 192 µg m^−3^, whereas 169 µg m^−3^ was measured in LEP. Considering these maximum values, there were no O_3_ concentrations above the alert threshold (240 µg m^−3^) during this period, while the information level threshold (180 µg m^−3^) was only exceeded during the study period twice in ASO: 29/06/2015 (15:00–16:00) with 192 µg m^−3^ and 16 June 2017 (14:00–15:00) with 181 µg m^−3^. Regarding the limit for the protection of human health (120 µg m^−3^), on average, and during this period, the number of days exceeding this threshold level was 51 year^−1^ and 18 year^−1^ in ASO and LEP, respectively, which clearly remarks differences in the location of both monitoring sites within the city. 

**Figure 3:**
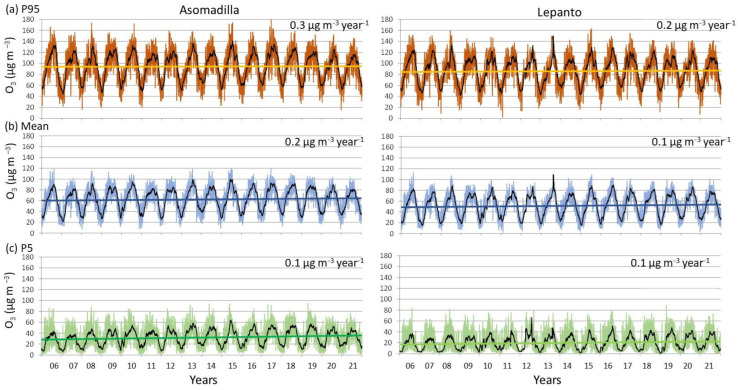
Temporal evolution for (**a**) daily 95th percentile, (**b**) mean and (**c**) 5th percentile of ozone from 2006 to 2021 at ASO and LEP, and the corresponding linear trend. One-month running average values for each time series are also shown.

Daily averages, and daily 95th and 5th percentiles, all of them obtained from hourly data, were used to analyse the O_3_ trends for the whole period studied (2006–2021), following the methodology applied in [16]. Figure 3 provides trend estimations based on daily means and daily 5th and 95th percentiles. It is necessary to mention that *p*-values were calculated to determine whether a trend is statistically significant using a 95% confidence level. To obtain the *p*-value, the Student’s t-test was applied. All *p*-values were below this threshold (95%), i.e., the trend is not statistically significant. However, we report all of these *p*-values following the recommendations performed in [20]. For daily averages, both sites presented a positive trend of 0.18 µg m^−3^ year^−1^ (*p* = 0.59) and 0.15 µg m^−3^ year^−1^ (*p* = 0.59), while the daily 95th percentiles showed a positive and higher trend (0.27 µg m^−3^ year^−1^ (*p* = 0.73) and 0.24 µg m^−3^ year^−1^ (*p* = 0.70)) than daily P5 (0.10 µg m^−3^ year^−1^ (*p* = 0.49) and 0.07 µg m^−3^ year^−1^ (*p* = 0.46)). These results indicate that the daily, maximum (P95) and background (P5) O_3_ values increased at both sampling sites, with special emphasis in maximum concentrations. As is well known, the O_3_ trend can be the result of several factors, such as modifications in local (emissions, weather patterns) and synoptic scales (climate change, synoptic meteorological patterns). In this sense, the O_3_ concentrations in the coastal area of the southwestern Iberian Peninsula [16] suggest that changes in the temperature and pressure during 2000–2021 point to a clear trend towards anticyclonic and warmer conditions, which, along with the reduction of precursor emissions, would contribute to the increasing trend of O_3_.

### 3.2. Yearly, Seasonal and Daily Evolution

**Figure 4:**
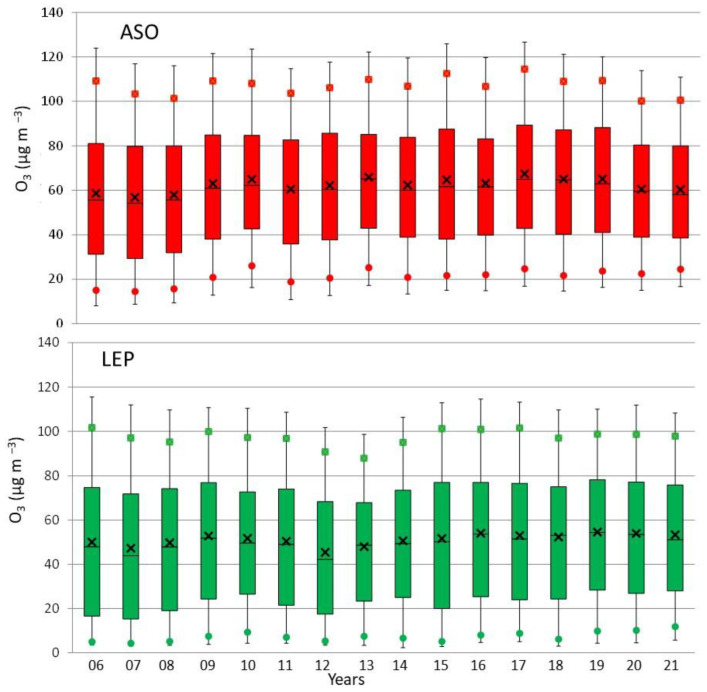
Box plots for ozone concentrations in ASO and LEP during the period 2006–2021. The centre of each box denotes the 50% (P50), and the bottom and top of the box correspond to the 25% (P25) and 75% (P75) values, respectively. The squares indicate the P90 and the circles the P10 values, while the extremes of the box represent the P95 and P5 values. The black cross indicates the average.

In this section, we illustrate the temporal variation of O_3_ in both sites at different temporal scales. As discussed above, the use of 16 years of data in this study allows the possibility to investigate the O_3_ year-to-year variability under different meteorological scenarios. This evolution is assessed in the corresponding box-and-whisker plots in Figure 4, which were calculated based on hourly data. Within this variability, it is interesting to note that the highest P95 value in ASO (126.6 µg m^−3^ in 2017) and the second one in LEP (113.2 µg m^−3^ in 2017) coincided with the highest occurrence of heat waves in the province (four events in 2017) [21], which clearly suggest a direct and positive influence of this synoptic scenario on high O_3_ concentrations. This result agrees with the conclusions of the recent analysis of the Copernicus Atmosphere Monitoring Service, in which temperature peaks are related to an increase in O_3_ levels [22].

In general, the distribution of O_3_ concentrations (P75-P25) was larger in LEP than in ASO. On average and for the whole period, this range was 51.5 µg m^−3^ and 46.0 µg m^−3^, respectively, with maximum ranges of 58.6 µg m^−3^ in LEP and 50.1 µg m^−3^ in ASO. On the contrary, the comparison also showed that the 50th percentile (P50) values from ASO were systematically higher than those from LEP. In this sense, the average of P50 during the whole period was 60.4 µg m^−3^ in ASO and 49.8 µg m^−3^ in LEP. Indeed, all percentiles calculated (Figure 4) were higher in ASO than in LEP, reinforcing the different urban environment in which each monitoring site is placed (Section 2). The largest differences between both were found for the smallest concentrations, which agree with the classification of each station (urban and suburban). LEP is more influenced by traffic density, while ASO is in a cleaner environment and is less directly affected by traffic emissions. 

**Figure 5:**
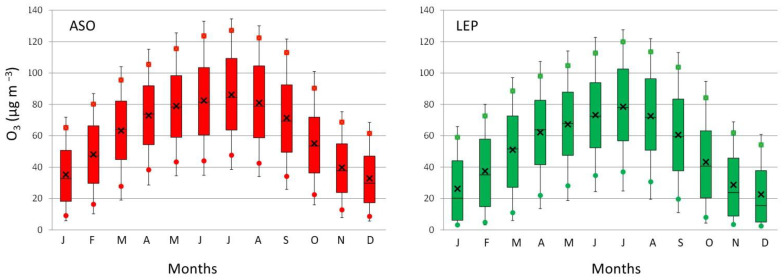
Monthly evolution of ozone concentration at Asomadilla and Lepanto sites (2006–2021).

As expected, the O_3_ showed both monthly and daily temporal variations at both monitoring stations. The monthly variability is shown in Figure 5, which clearly agrees with the seasonality suggested in Figure 2. The monthly variability showed that during the first months of the year and for both stations, there was a continuous increase in the monthly value of O_3_, reaching higher concentrations in the warm months (May–August) than in the cold ones (December–January). From April to September, the monthly means were over 60.0 µg m^−3^, reaching the monthly maximum in July, with an average of 86.2 µg m^−3^ and 78.5 µg m^−3^ in ASO and LEP, respectively. There was a large agreement in the occurrence of the maximum monthly concentrations between both stations (88% of the years). There were only two years (2008 and 2021) in which there was no coincidence in the monthly maximum between the sampling stations. While 2021 is the year after the strict COVID-19 lockdown in Spain, 2008 coincides with the beginning of the economic recession in Spain, in which an abrupt reduction in NO_2_ concentrations of about 7% per year from 2008 to 2012 was observed in densely populated cities in Spain [23]. 

Both stations presented a similar growth rate in the monthly average from December to July—6.6 µg m^−3^ in ASO and 6.9 µg m^−3^ in LEP—while the amplitude of the monthly cycle (the difference between the maximum and minimum) was similar at 54 µg m^−3^ in ASO and 55.9 µg m^−3^ in LEP. These monthly concentrations represent a balance between the generation–destruction processes of O_3_ and meteorological conditions on local, regional, and global scales [24]. While changes in the emissions within the city causes temporal variability in the presence of precursors during the year, this pattern also reacts to changes in the meteorological conditions, such as solar radiation and temperature increasing or limiting the photolytic cycle [2], and synoptic patterns and mesoscale circulations ventilating or accumulating air masses in the area [11,25].

**Figure 6:**
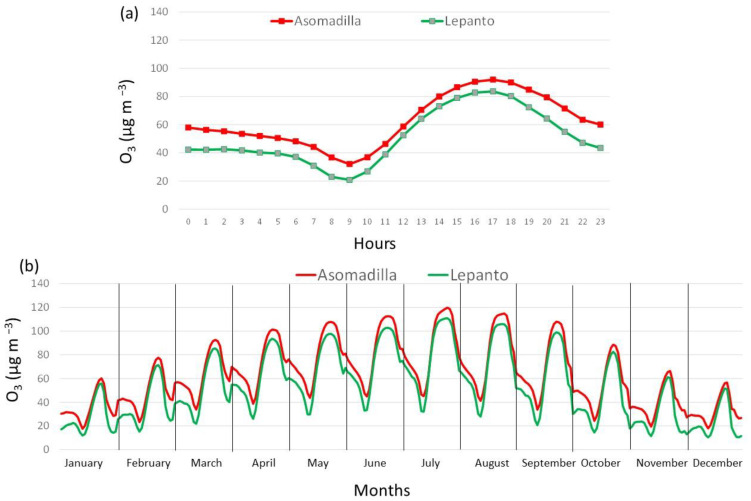
(**a**) Daily cycle and (**b**) monthly daily cycle in Asomadilla and Lepanto during the whole sampling period (2006–2021).

Figure 6a displays the daily cycle of hourly O_3_ concentrations at both sampling sites considering the whole sampling period (2006–2021). This figure shows that, on average, higher concentrations were recorded during the daytime than during the night, following the daily solar radiation cycle. It is well documented that solar radiation is one of the main factors in understanding the diurnal variability of O_3_ [26,27] due to O_3_ being a secondary photochemical pollutant generated by the photochemical reaction between NO_2_ and VOCs. The O_3_ concentrations gradually increase just after sunrise, which is associated with the increase in the convective activity in the planetary boundary layer causing the collapse of the nocturnal inversion layer. This fact favours the down-mixing processes of the previous day’s O_3_ from the reservoir layer, and photochemical reactions with NOx and VOCs, explaining the observed progressive increase from mid-morning to noon. Low concentrations of ozone at night are attributed primarily to the lack of photochemical reactions [28]. The lowest concentrations are registered in LEP, while the highest ones in ASO, coinciding in time, i.e., the highest concentration (91.9 µg m^−3^ and 83.6 µg m^−3^) at 17:00 UTC, and the lowest (32.0 µg m^−3^ and 20.8 µg m^−3^) in the early morning at 9:00 UTC, around sunrise. Differences in the intensity of the daily values observed between both stations reflect the combination of different factors, such as the precursor emissions, and chemical and physical processes [29,30]. In this sense, differences in the urban environment (Section 2) influence the intensity of the minimum concentrations, for instance, favouring reactions between O_3_ and NO_2_, which results the formation of dinitrogen pentoxide (N_2_O_5_) and nitric acid (HNO_3_) [31]. 

This daily evolution was observed during the whole year, although with differences in intensity and in the hours of maximum values. Figure 6 displays the monthly variability of the daily cycles at both sampling stations. The maximum values were in ASO and LEP from 119.6 µg m^−3^ and 110.8 µg m^−3^ in July to 56.3 µg m^−3^ and 51.7 µg m^−3^ in December, respectively, while the minimum values were in ASO from 45.2 µg m^−3^ in July to 17.9 µg m^−3^ in December, while in LEP were from 32.9 µg m^−3^ in June to 10.3 µg m^−3^ in January. The time occurrence of the maximum and minimum values also presented a seasonal variability. The daily maximum was registered between 18:00 and 19:00 UTC in summer in ASO and LEP, but in winter it was observed at 17:00 UTC in ASO and between 16:00 and 17:00 UTC in LEP. On the contrary, the minimum was at 9:00 UTC in ASO and between 8:00 and 9:00 UTC in LEP. In agreement with previous work [32], during the warm months, the O_3_ diurnal cycle showed high values between 12:00 and 18:00 UTC. This figure also shows monthly differences in the maximum and minimum values between the sites. While both stations present similar maximum values and more differences in the minimums in winter and autumn, there are many more differences in the maximum and more similarities between the minimum values in summer and spring. In the case of the minimum values, the combination of limited mixing in the nocturnal boundary layer, dry deposition, and nitric oxide titration help to reach the minimum values in LEP, and more differences with those minimum values were registered in ASO in winter. The occurrence of higher maximum values in ASO in summer and spring, on the contrary, can be associated with local photochemical production and with the transport of O_3_ precursors to areas a bit out of the city (suburban).

### 3.3. Impact of the COVID-19 Lockdown on O_3_ Surface Concentrations

In the present section, we investigate the variation in the O_3_ concentrations in both monitoring stations in the context of COVID-19 disease. On 14 March 2020, a state of alarm was declared in Spain due to the COVID-19 outbreak (Real Decreto 463/2020). The strict lockdown started on 16 March and ended on 2 May. During this period, all non-essential workers were ordered to remain at home and only the supply of food and products necessary for public health was guaranteed. In a general context, while the impact of this lockdown had enormous repercussions for individuals and communities, one of the most positive impacts was the reduction in pollutants and greenhouse gases, i.e., primary atmospheric pollutants reduced by 50% [33].

**Figure 7:**
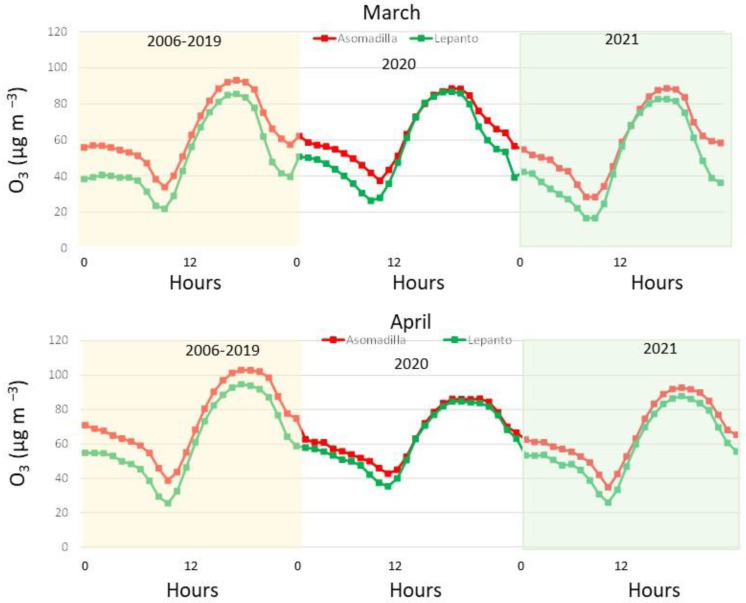
Daily cycles in March and April calculated for the whole period (2006–2019) for 2020 and for 2021 in Asomadilla and Lepanto.

Considering the strict lockdown period, we focus on two months, March and April, and we have compared the O_3_ concentrations in these months across three different periods, from 2006 to 2019, in 2020, and in 2021, one year after the lockdown. Figure 7 displays the corresponding daily cycles of March and April in ASO and LEP for each period considered. On average, the O_3_ concentrations (2006–2019) in both stations ranged from 41.3 µg m^−3^ (in 2012) to 72.0 µg m^−3^ (in 2019) in March and from 55.3 µg m^−3^ (2012) to 82.5 µg m^−3^ (2017) in April. These values were in line with those calculated in March 2021 (58.7 µg m^−3^ in ASO and 48.2 µg m^−3^ in LEP) and in April 2021 (66.5 µg m^−3^ in ASO and 59.4 µg m^−3^ in LEP). The averages obtained at both stations in 2020 reveal a different impact of the lockdown period. In March, the averages were higher at both stations compared to the 2006–2019 average (O_3_ increment of 0.5% in ASO and 11% in LEP), while in April, the O_3_ concentrations drastically decreased in ASO (−11%) and it was similar to the 2006–2021 average in LEP (0.03%). Hence, these results indicate that there was not a large decline in O_3_ concentrations in Córdoba city compared to the 2006–2019 average. However, and taking as reference the averages of March and April in 2019 and 2020, a large influence of the lockdown is well observed, with a reduction in March of 7.8% in ASO and 1% in LEP, while in April, it was of 12% in ASO and 8% in LEP. This can be associated with the large decrease in the anthropogenic NOx emissions during the lockdown. The authors in [34] reported that the reductions were at least 15% globally and 18% to 25% regionally in April and May 2020, while urban emissions were reduced in Spain up to 50% or 60% [35]. 

Following the methodology applied in [19], we have calculated the averages between 22–09 h and 12–18 h for each period to quantify the differences between the background values and local–regional O_3_ production. The averages and differences are shown in Table 1. These results revealed that the regional-to-hemispheric O_3_ background (averages from 22–9 h) only decreased in April 2020 in ASO with respect to historic data, from 62.1 to 56.5 µg m^−3^, while there was a decrease in the local diurnal O_3_ formation at both stations, being more intense in April (from 91.9 to 79.3 µg m^−3^ in ASO and from 84.1 to 77.9 µg m^−3^ in LEP). These values revealed that the diurnal O_3_ formation (DOF) (difference between averages 22–09 h and 12–18 h) decreased in March and especially in April 2020, while in 2021, the numbers were similarly reversed with respect to 2020.

These results indicate that the strict lockdown mainly impacted the production of O_3_ in Córdoba city, while the background values presented similar or even higher values. These results do not agree with those observed in 15 regional background sites in the Iberian Peninsula [19] since at these sampling sites, the hemispheric regional O_3_ background was significantly reduced during the health crisis. This fact, in a general framework of a decrease in anthropogenic emissions over the Iberian Peninsula [35], and hence, fewer precursors available, pointed to the large variability in O_3_ concentrations and the different impact of this lockdown on the sampling stations, according to their location and environment. 

## 4. Conclusions

In this work, the temporal and spatial variations of the O_3_ concentrations measured at two sites in Córdoba city were characterised. For this purpose, 16 years of O_3_ concentrations at the Asomadilla (suburban) and Lepanto (urban) stations were used. The hourly mean concentrations of O_3_ in Asomadilla were 62.0 µg m^−3^, while they were 51.3 µg m^−3^ in Lepanto. Only twice did the maximum hourly concentrations exceed the value of 180 μg m^−3^ during this period. The analysis of trends presented a positive trend, between 0.07 and 0.27 µg m^−3^ year^−1^ for daily means, and daily P95 and P5, registering the highest annual variability in daily P95 concentrations in both stations. The monthly means showed a clear seasonal cycle, with the maximum levels in July and the minimum levels in December, while the daily cycle presented minimum values in the early morning (9:00 UTC) and maximum values in the afternoon (17:00 UTC), with differences according to the time of the year and the measurement site. In this work, we have also demonstrated that the impact of the COVID-19 lockdown was mainly registered in April 2020, with a decline in O_3_ concentrations of over 10%. This reduction was mainly associated with the decrease in the local O_3_ production (average of hourly concentrations between 12 and 18 h). These results will allow the possibility to perform future analyses to investigate, for instance, the impact of local and synoptic meteorological conditions on O_3_ concentrations, and the influence of heatwave events.

## Figures and Tables

**Table 1:** Differences in O_3_ concentrations in March and April considering three periods (2006–2019, 2020, and 2021). DOF means diurnal.

Ozone Formation.					
		March		April	
		ASO	LEP	ASO	LEP
2006–2019	22–9 h	51.9	35.7	62.1	48.0
	12–18 h	83.7	75.6	91.9	84.1
	DOF	31.9	39.9	29.8	36.0
2020	22–9 h	53.9	42.6	56.5	51.5
	12–18 h	80.7	79.6	79.3	77.9
	DOF	26.8	37.1	22.8	26.5
2021	22–9 h	46.6	31.7	55.6	46.8
	12–18 h	79.5	75.2	84.5	79.3
	DOF	32.8	43.5	28.9	32.4
